# Free thiol groups on poly(aspartamide) based hydrogels facilitate tooth-derived progenitor cell proliferation and differentiation

**DOI:** 10.1371/journal.pone.0226363

**Published:** 2019-12-19

**Authors:** Orsolya Hegedűs, Dávid Juriga, Evelin Sipos, Constantinos Voniatis, Ákos Juhász, Abdenaccer Idrissi, Miklós Zrínyi, Gábor Varga, Angéla Jedlovszky-Hajdú, Krisztina S. Nagy

**Affiliations:** 1 Department of Oral Biology, Semmelweis University, Budapest, Hungary; 2 Laboratory of Nanochemistry, Department of Biophysics and Radiation Biology, Semmelweis University, Budapest, Hungary; 3 University of Lille, Faculty of Science and Technology, Villeneuve d’Ascq Cedex, France; Michigan Technological University, UNITED STATES

## Abstract

Cell-based tissue reconstruction is an important field of regenerative medicine. Stem and progenitor cells derived from tooth-associated tissues have strong regeneration potential. However, their in vivo application requires the development of novel scaffolds that will provide a suitable three-dimensional (3D) environment allowing not only the survival of the cells but eliciting their proliferation and differentiation. Our aim was to study the viability and differentiation capacity of periodontal ligament cells (PDLCs) cultured on recently developed biocompatible and biodegradable poly(aspartamide) (PASP)-based hydrogels. Viability and behavior of PDLCs were investigated on PASP-based hydrogels possessing different chemical, physical and mechanical properties. Based on our previous results, the effect of thiol group density in the polymer matrix on cell viability, morphology and differentiation ability is in the focus of our article. The chemical composition and 3D structures of the hydrogels were determined by FT Raman spectroscopy and Scanning Electron Microscopy. Morphology of the cells was examined by phase contrast microscopy. To visualize cell growth and migration patterns through the hydrogels, two-photon microscopy were utilized. Cell viability analysis was performed according to a standardized protocol using WST-1 reagent. PDLCs were able to attach and grow on PASP-based hydrogels. An increase in gel stiffness enhanced adhesion and proliferation of the cells. However, the highest population of viable cells was observed on the PASP gels containing free thiol groups. The presence of thiol groups does not only enhance viability but also facilitates the osteogenic direction of the differentiating cells. These cell-gel structures seem to be highly promising for cell-based tissue reconstruction purposes in the field of regenerative medicine.

## Introduction

Stem cells (SC) and stem cell-like progenitor cells have unequivocally great regenerative potential that presumably could revolutionize regenerative medicine. These cells can be obtained from either embryonal or postnatal tissues. However, the application of embryonal stem cells in the clinical practice is still a highly debatable point due to the ethical and technical issues involved [1]. Therefore, postnatal and adult stem cells are currently in the focus of research in the field of regenerative medicine[2].

The first postnatal stem cells with multipotent differentiation capacity were discovered in the bone marrow in 1966 [3]. Since then, such cells have been successfully obtained from various tissues, even from tooth-associated ones [4]. Tooth-derived stem and progenitor cells can be easily isolated from patients via a minimally invasive procedure. In addition, their proliferation and differentiation potential is comparable to bone marrow stem cells [4]. Dental stem and progenitor cells can be obtained from different tissues of the teeth, including that from the most noninvasively accessible one: the periodontal ligament. Cultures of periodontal ligament (PDL) cells have been demonstrated to contain stem cell-like cells and undergo osteogenic, chondrogenic and adipogenic differentiation in vitro while exhibiting the ability for periodontal fiber and bone regeneration in vivo [5–8]. In previous studies of our research group, their capability for neurogenic differentiation [9] as well as their immunomodulatory effects were also described [10]. These advantageous features further support the importance of PDL-derived cell cultures in future applications in regenerative medicine or dentistry. Nevertheless, tissue regeneration therapies require appropriate biocompatible and biodegradable scaffold materials which are able to provide optimal conditions for the cells during and after transplantation [11]. One of the main focus points of tissue engineering strategies is to develop artificial scaffolds closely resembling the physiological environment of the cells, namely the extracellular matrix (ECM). Apart from supporting cell survival, an ideal scaffold must also facilitate cell adhesion, proliferation and migration. Furthermore, scaffolds should be designed with similar physical, chemical and biological properties to the ECM, allowing not only effective implantation but delivery of growth factors, nutrients and oxygen as well [11]. As different cell types favor different conditions for their growth and/or differentiation, the above-mentioned properties should be customized according to the requirements of each cell type[12].

Building of naturally existing functionalities into the scaffolds which can covalently bind the often occurrent functional groups of membrane proteins like amino (NH2) or thiol groups (SH) is a widely used approach in regenerative medicine. Reduced thiols on the surface of the mammalian cell play an important role in protection against oxidating agents and also take part in cell signaling [13]. Since thiol groups are able to form disulphide bonds *in vivo*, thiol functionalized polymers can be crosslinked or bound to cell surface proteins under physiological conditions [14, 15]. Free SH groups of cysteine on scaffolds can be used for binding thiol-reactive small or large molecules to install wide range of functions as cysteine is frequently present in regulatory, catalytic or binding site of various natural proteins [16, 17]. Besides, thiol groups also have positive effect on cell behavoiur via enhancing the surface potential of the scaffolds and providing additional adhesion sites for the cells [18]. Moreover, significance of the thiol groups in adhesion of urinary bladder mucosa [19] and marine mussels [20] was also demonstrated.

Hydrogels undoubtedly belong to the most promising scaffolds for regenerative therapies since they have physicochemical properties similar to natural tissues [21]. In addition, their chemical composition, structure and mechanical properties can be tailored in a broad range by various modifications in order to meet specific prerequisites according to the intended applications [11, 22, 23]. Hydrogels are unique materials whose intrinsic properties allow them to behave as both liquid (nutrient can diffuse through them) and solid materials (maintaining their own structures while deformable to external forces as soft materials). Moreover, some of these intelligent materials are also able to respond to the changes in such physiologically relevant parameters of the environment, such as temperature, pH or presence of certain bioactive molecules [24–26]. Furthermore, in hydrogels fabricated for biomedical applications, cell migration through their well-defined porous structure has also been demonstrated [27, 28].

Currently used hydrogel scaffolds are based on either natural polymers such as polysaccharides (e.g. dextran, cellulose) or synthetic ones such as poly(esther)s, poly(urethane), poly(esther amide)s and poly(ether)s [29, 30]. Since the ECM contains large amounts of proteins (e.g. collagen, fibronectin), it is most possible that poly(amino acid) based materials are suitable for application in stem cell therapy due to their chemically similar structures [31, 32]. However, poly(amino acid) synthesis is rather expensive and technically difficult thus the investigation of poly(amino acid) based chemically cross-linked hydrogels as scaffolds is limited [18, 33–37].

The aim of this study is to develop novel poly(aspartamide) based hydrogels that are suitable as scaffolds for in vitro cell culturing of tooth-derived cells. The applied hydrogel types were chosen according to our previous studies where an osteosarcoma cell line (MG-63) was cultured on poly(aspartamide) gels with different crosslinkers. One of the crosslinkers (diaminobutane) is stable under physiological conditions while the other one (cystamine) is biologically active and provide dynamic behavior and free thiol groups in the polymer matrices. Based on our results, free thiol groups in the gel seem to facilitate the adhesion and proliferation of osteoblast-like cells [34]. In this paper, the effect of physical and chemical properties of these gels on adhesion, proliferation and osteogenic differentiation of PDLCs was comprehensively investigated with a particular emphasis on the amount of thiol groups incorporated into the gels.

## Materials and methods

### Materials

L-aspartic acid (Sigma-Aldrich, UK), 1,4-diaminobutane (DAB) (Sigma-Aldrich, ≥99%), cysteamine (CYSE) (Sigma-Aldrich, UK), cystamine (CYS) (Sigma-Aldrich, UK), dimethylformamide (DMF) (VWR International, USA), dimethylsulfoxide (DMSO) (Sigma-Aldrich), o-phosphoric acid (VWR), imidazole (ACS reagent, ≥99%, Sigma-Aldrich), citric-acid*H_2_O (ACS reagent, ≥99.9%, VWR), sodium chloride (99–100.5%, Sigma-Aldrich), phosphate buffer saline (PBS) (Tablet, Sigma), D,L-dithiotreitol (DTT) (Sigma), 5,5 dithio bis-(2-nitrobenzoic acid) (Sigma, ≥98%, USA), L-cystein (Sigma, ≥97%, USA), Humidified incubator (Nuaire, USA), 100 mm tissue culture dishes (Orange Scientific, Belgium), 48 well plates (Sigma-Aldrich, USA), low cell binding 96 well plates (Nunc, Denmark), Eagle’s Medium Alfa minimal essential medium (αMEM) (Gibco, USA), fetal bovine serum (FBS, Gibco, USA), L-glutamine (Gibco, USA), penicillin and streptomycin (Gibco, USA), L-ascorbic acid 2-phosphate (Sigma-Aldrich, USA), beta-glycerophosphate (Sigma-Aldrich, USA), dexamethasone (Sigma-Aldrich, USA), WST-1 [2-(4-Iodophenyl)-3-(4-nitrophenyl)-5-(2,4-disulfophenyl)-2H-tetrazolium] (Roche, Switzerland), Vybrant DiD (Molecular Probes, USA), 2-Amino-2-Methyl-1-Propanol buffer (Sigma-Aldrich, USA), Alkaline Phosphatase Yellow (pNPP) Liquid Substrate System (Sigma-Aldrich, USA).

### Preparation of poly(succinimide)

Poly(succinimide) (PSI) was synthesized by thermal polycondensation of L-aspartic acid in the presence of o-phosphoric acid at high temperature under vacuum. 20 g L-aspartic acid and 20 g o-phosphoric acid were mixed in a 1 L glass flask then transferred to a rotary evaporator. The reaction mixture was heated to 180 °C while the pressure was progressively reduced to finally reach 5 mbar. After the reaction sequence, the polymer was dissolved in DMF. This step was followed by obtaining the precipitation and washing it with water until reaching neutral pH. The polymer was subsequently dried at 40 °C for 2 days. The yield of the reaction was between 95 and 99% and the viscosity-average molecular mass (Mη) was 28.5 ± 3 kDa. The determination of the molar mass and the synthesis were described in detail in our previous publications [34, 35].

### Synthesis of different poly(aspartamide) hydrogels

#### Preparation of poly(succinimide) gels with different cross-linkers and cross-linking ratio

Two different diamines, namely 1,4-diaminobutane (DAB) and cystamine (CYS) were used to prepare PSI based gels. The samples were synthesized according to the same process and with an identical chemical constitution as described in our previous study [34]. The amount of the applied chemicals can be found in S1 Table. When cross-linking was introduced, the concentration of the PSI in the reaction mixture (cp) was 15 wt % in each sample. The degree of crosslinks (D_cr_) was calculated from the molar ratio of the cross-linkers (n_cr_) to the monomer units (n_m_) according to the following formula:

$$D_{cr}=\frac{n_{cr}}{n_{m}}$$ (1)

Apart from the two gel types where either DAB (PSI-DABD_cr_) or CYS (PSI-CYSD_cr_) were used as a crosslinker, a third gel type was also synthesized with a 1:1 molar ratio of DAB and CYS (PSI-CYS-DABD_cr_). All these hydrogels were assembled with different cross-linking degrees (D_cr_ = 1/10, 1/20, and 1/40). The chemical and mechanical characterizations as well as the biodegradability studies of these gels were performed and published in our previous work [34].

#### Preparation of poly(succinimide) gels with different thiol contents

To prepare PSI gels with different thiol concentrations, PSI was modified with different amounts of cysteamine (CYSE). In these experiments, DAB was used as the cross-linker with constant cross-linking ratio (D_cr_ = 1/20). DAB and CYSE were dissolved in dimethyl-sulfoxide (DMSO) then thoroughly mixed with a 25 wt% PSI/DMSO solution. Therefore the modification and the cross-linking reaction occurred simultaneously. The mixture was subsequently transferred to a 0.75 mm thick glass frame. The constitution of the reaction mixture can be seen in S2 Table. After 24 hours, the gels were removed from the mold and were eventually immersed in DMSO to wash the non-reacted molecules. The cross-linking reaction can be seen on S1 Fig Step 1. The samples were classified by the molar ratio of the CYSE (n_CYSE_) to the monomer units (n_m_), in the same way as described in the previous section. The thiol content (D_SH_) can be calculated with the following equation:

$$D_{SH}=\frac{n_{CYSE}}{n_{m}}$$ (2)

The gels were prepared with different D_SH_ (D_SH_ = (1/2), (1/5), (1/10), (1/20), (1/40) and (1/80)). When gelation was introduced, the c_p_ was 15wt % in each sample.

#### Synthesis of the poly(aspartamide) hydrogels

The poly(aspartamide) (PASP) gels were prepared from PSI gels by mild alkaline hydrolysis. The gels were immersed in an imidazole-based pH = 8 buffer (I = 0.25 M, c = 0.1 M) to open the succinimide rings but avoid the cleavage of their disulphide bonds as well as the amide bonds between the cysteamine and the polymer chain. The buffer was changed daily for 4 consecutive days to progressively remove the DMSO and every unreacted molecule. The reaction can be seen in S1 Fig Step 2.

#### Cleavage of the disulphide bonds in the hydrogels

In order to generate free thiol groups in the PASP based hydrogels, gel samples (PASP-CYSE_(X)_-DAB_1/20_, PASP-CYS-DAB_1/10_ and PASP-CYS-DAB_1/20_) were immersed in 0.1 M dithiotreitol (DTT)/pH = 8 buffer solution. The reaction can be seen in S1 Fig Step 3. Due to the disulphide cleavage, the degree of crosslinks doubled so the D_cr_ of the 1/10 samples became 1/20 (CYS-DAB_1/10_ turned to CYSE-DAB_1/20_) and that of the 1/20 samples became 1/40 (CYS-DAB_1/20_ turned to CYSE-DAB_1/40_). During gelation, the thiol groups in CYSE could be oxidized to disulphide bridges, therefore these samples were treated with DTT as well. The DTT solution was changed twice during the following 2 days to cleave every disulphide bond in the hydrogels. After the reduction of the disulphide bridges, the gels were washed several times with phosphate buffer saline (PBS, pH = 7.4, I = 0.15 M), the solution which was used during the cell experiments.

### Determination of the thiol group concentration

Spectrophotometry was utilized to assess the presence and quantity of thiol group concentrations in different hydrogels. The measurements were carried out before and after the DTT treatment of the PASP CYSE_(X)_-DAB hydrogels. Before the determination, the samples were washed with ultra-pure water to remove the unbound chemicals, such as buffer compounds or excess amount of DTT, from the gel matrices. After thorough washing, the gels were freeze-dried to avoid the formation of disulphide bridges. The exact amount of thiol groups in the gel samples was determined with Ellmann’s reagent according to the method published by Gyarmati et al. [38]. Dried gel samples weighing 10 mg were dropped into 1.8mL reagent buffer and 20–20 μL Ellman’s reagent solution was added to the mixtures. After a 4-hour-long incubation, light absorbance was measured at 455 nm using an Agilent 8453 spectrophotometer. For calibration, L-cystein was used between 0 0.004 mmol range (S2 Fig).

### Determination of the chemical structure of the hydrogels by FT-Raman spectroscopy

Raman spectra of the hydrogel samples were obtained at an ambient temperature using a LabRam HR visible micro-Raman spectrometer (produced by HORIBA Jobin Yvon, France) equipped with a confocal microscope (50x magnifying objective lens was used), in back-scattering geometry in the spectral range 200–4000 cm^−1^. A He-Ne laser (λ = 632.81 nm) was used for excitation. The applied laser power was 120 mW. The Raman signal was collected with a CCD-detector (1024 x 256 pixels) placed after a diffraction grating (1600 grooves/mm) with a final spectral resolution of 2 cm^−1^. Spectra were obtained from 1000 scan with an exposure time of 60 s for each orientation of the grating which provided the spectra with a good signal to noise ratio.

### Scanning electron microscopic (SEM) analysis of the hydrogels

To examine the 3D structure and determine the porosity of the hydrogels, SEM analysis was carried out. After the DTT treatment, hydrogels were thoroughly washed with ultrapure water and subsequently freeze dried. Microscopic pictures were taken using a ZEISS EVO 40 XVP scanning electron microscope equipped with an Oxford INCA X-ray spectrometer (EDS). The applied accelerating voltage was 20 kV. The samples were fixed on a special conductive sticker with tweezers. Before examination, samples were sputter coated with palladium in a thickness of 20–30 nm with a 2SPI Sputter Coating System. The average pore size of the samples was determined by the Fiji (ImageJ) image analysis program with 20 parallel measurements.

### Isolation and cultivation of tooth-derived cells

Cells were obtained from the connective tissue adjacent to human wisdom teeth. The teeth were surgically removed from healthy young adults in the Department of Dentoalveolar Surgery (Semmelweis University, Budapest, Hungary). We have obtained written consent from each patient. This study was approved by the Semmelweis University Regional and Institutional Committee of Science and Research Ethics. The number of the ethical permission is: 17458/2012/EKU.

Periodontal ligament cells were then isolated according to our already established protocol [9] and maintained in a humidified incubator under standard culture conditions (37 °C, 5% CO_2_, 100% humidity). PDLCs were cultured in alpha modification of Eagle’s Minimal Essential Medium Alfa (αMEM) and supplemented with 10% fetal bovine serum, 2 mM L-glutamine, 100 units/ml penicillin and 100 mg/ml streptomycin.

### Cell viability assay using WST-1 reagent

To measure cell viability, gel discs with a diameter of 3 mm were prepared and placed in low cell binding 96 well plates (Nunc, Denmark). 20 000 PDLCs in 200 μl culture medium were seeded on each gel disc. After culturing for 1, 3, 7 and 14 days, cell viability was assessed utilizing the WST-1 cell proliferation reagent according to our previously published protocol [34] using a microplate reader (Model 3550, Bio-Rad Laboratories, Japan) at 450 nm with the reference wavelength of 650 nm. Gel discs without cells were used as negative controls.

### Phase contrast and two photon microscopic studies

For the phase contrast microscopic investigation, gel discs with a diameter of 5 mm were prepared and placed in 48 well plates. Then 40 000 PDLCs in 400 μl culture medium were seeded on each gel disc. After 1, 3, 7 and 14 days, cell morphology was observed under a phase contrast microscope (Nikon Eclipse TS100, Nikon, Japan). Microphotographs were taken by a high performance CCD camera (COHU, USA) applying Scion image software. In order to make the cells visible under a two photon excitation microscope (Femto2d, Femtonics, Hungary), PDLCs were labeled with a vital dye, Vybrant DiD (Molecular Probes, USA) before seeding them on the gel discs. After 1, 3, 7 and 14 days, the samples were washed in PBS (37 °C) and fixed with 4% paraformaldehyde solution at room temperature (RT) for 2 hours. After that, the fixed samples were washed twice then stored in PBS at 4 °C until the examination. To excite the fluorescent dye, a Spectra Physics Deep See laser was used at 800 nm wavelength. Images were taken under a 10 x objective by the MES4.4v program. The contrast and brightness of the images were adjusted by the Fiji (ImageJ) image analysis program.

### Measurement of alkaline phosphatase (ALP) activity

For detecting the expected osteogenic differentiation of the cells growing on the different gel types, ALP enzyme activity was assessed on the 3^rd^, 7^th^ and 14^th^ day after the osteogenic induction. The composition of the osteogenic medium was previously described [9]. To measure the ALP activity of the PDLC cultures, the cells were first lysed in a 2-amino-2-methyl-1-propanol buffer then to, the Alkaline Phosphatase Yellow (pNPP) Liquid Substrate System was utilized according to our previously published protocol [9]. Control samples were maintained in normal growth medium throughout the whole experiment. Gel discs without cells were used as negative control. Changes in the color were detected by measuring absorbance at 405 nm applying a microplate reader (Model 3550, Bio-Rad Laboratories, Japan).

### Statistical analysis

To determine cell viability and ALP activity, PDLC cultures derived from at least 3 different patients were used. In case of each tooth sample, 5 parallel measurements were performed for each experimental group, so the arithmetic mean values displayed on the diagrams were calculated from 15–25 independent experimental data. Statistical evaluation of the data was carried out by STATISTICA 10 software applying the Kruskal-Wallis non-parametric ANOVA followed by a median test. A difference was considered as statistically significant if p < 0.05.

## Results and discussion

In the past few years, several studies attempting to create an ideal scaffold for tooth-derived stem cells were published [39, 40]. Notwithstanding, only a few research papers appeared in the literature on PASP-based hydrogels, moreover most of them mainly focused on drug delivery [41–44]. These PASP-based hydrogels seem to be excellent candidates to culture cells, since they can mimic the chemical and mechanical properties of the ECM as it was demonstrated in our previous work using a tumor cell line [34]. However, the aim of our current study was to find the ideal gel composition that would provide suitable conditions for culturing and differentiating periodontal ligament-derived cells in order to be subsequently used for in vivo preclinical tests and eventually for developing regenerative therapies.

### Chemical and and physical characterization of the PASP-CYSE_X_-DAB_1/20_ hydrogel

**Fig 1:**
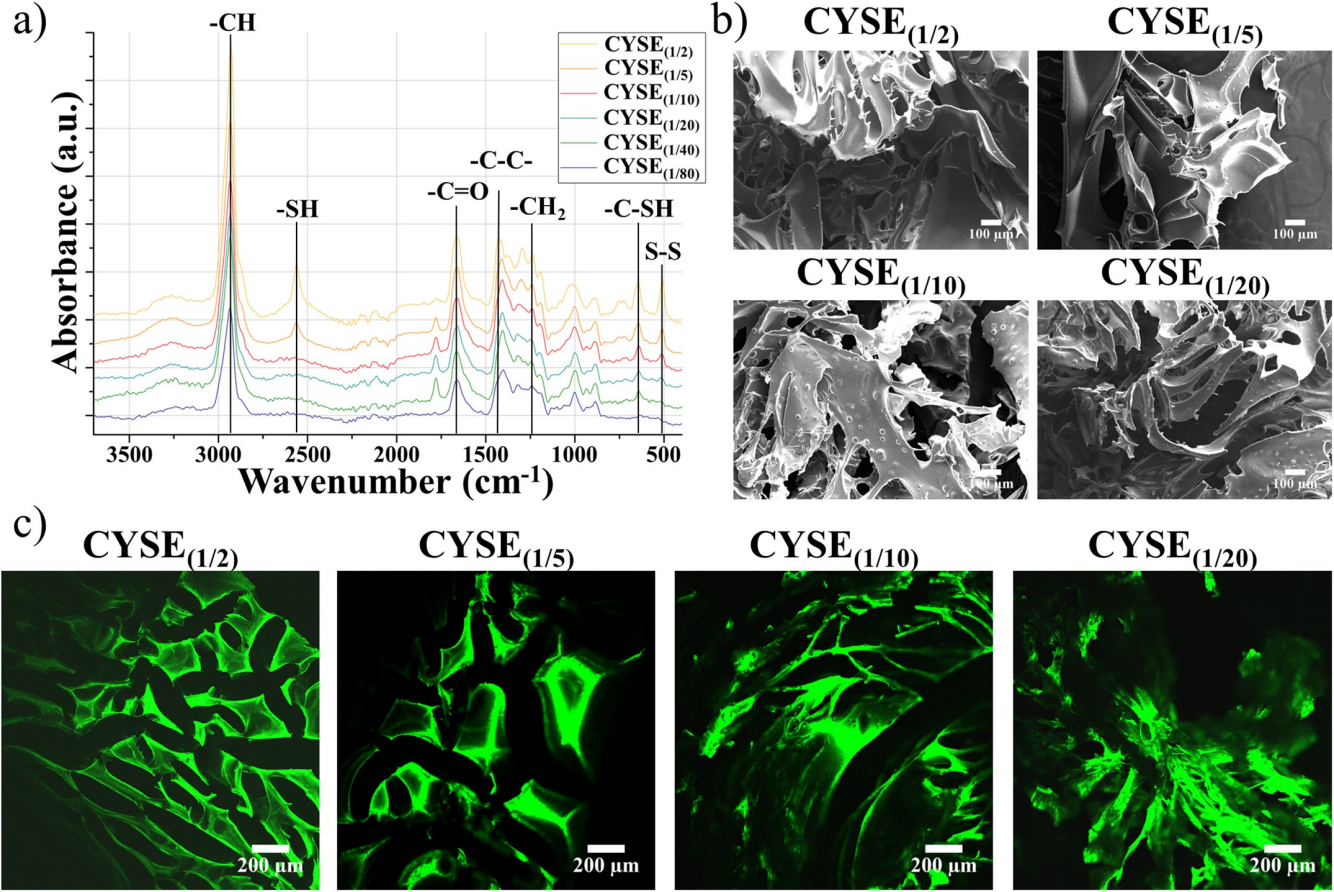
FT-RAMAN spectra of the dried hydrogels (a), SEM (b) and two photon images (c) of the freeze dried hydrogel matrices.

The chemical structure of PASP-CYSE_(X)-_DAB hydrogels (S1 Fig) was investigated by FT-RAMAN spectroscopy while the physical structure and the porosity of the hydrogels were assessed by SEM and two photon microscopies (Fig 1). The only changing parameter in the hydrogels is the free thiol content, thus we are using only the CYSE_(X)_ to indicate the differences between the hydrogel samples.

On the RAMAN spectra of the hydrogels (Fig 1a), the characteristic peaks of the succinimide and aspartamide can be found on each spectrum at 2930 cm^-1^ (-CH groups) and between 1200–1500 cm^-1^ (-CH2 and -C-C- groups), respectively. The peaks at 1662 and 1780 cm^-1^ indicate the vibration of the −C = O groups in the aspartic acid. The peak at 643 cm^-1^ is connected to the C-S bond in the cysteamine. The intensity of this peak is decreasing as the amount of cysteamine applied during the synthesis is increasing, then finally completely disappears in case of the CYSE_(1/80)_ samples. At 509 cm^-1^ S-S bonds appear with the increasing thiol content, although the samples were treated with DTT. This re-formation of disulphide bridges can be the result of thiol group oxidation during the drying of the gels. The characteristic peaks of the thiol groups can be seen at 2561 cm^-1^. The decreasing intensity of the thiol peaks further suggests the oxidation of the thiol groups during the drying process. To quantify the amount of the thiol groups, the Ellman’s reagent was utilized. As the results summarized in Table 1 show, there are no free thiol groups in the gel matrices before the DTT treatment. Therefore, all of the thiol groups in the cysteamine were oxidized into disulphide bridges. After DTT treatment and drying, the amount of the thiol groups increased, although lower values were attained than the ones theoretically expected and calculated due to the oxidization of the thiol groups during the drying process. At higher concentration of thiol groups, more thiol groups can be oxidized back to disulphide bonds which leads to the small difference of the measured values in the case of CYSE_(1/2)_, CYSE_(1/5)_ and CYSE_(1/10)_ samples, however the differences in swollen state are significantly higher.

**Table 1:** Amounts of the thiol groups in a 10 mg dried gel sample before and after DTT treatment and by theoretical calculation.

	CYSE _(1/2)_	CYSE _(1/5)_	CYSE _(1/10)_	CYSE _(1/20)_	CYSE _(1/40)_	CYSE _(1/80)_
Before DTT (μmol)	0.02 ± 0.02	0.04± 0.03	0.04± 0.02	0.03± 0.01	0.05± 0.02	0.02± 0.01
After DTT (μmol)	1.95± 0.04	1.41± 0.05	1.71± 0.08	0.30± 0.02	0.16± 0.02	0.02± 0.01
Theoretical (μmol)	18.6	7.4	3.7	1.85	0.92	0.46

On the SEM images (Fig 1b), the average pore size of the gels is found to be around 70–80 μm (Table 2), which is remarkably higher than the size of the PDLCs (which is around 30–40 μm). Poly(lactic-co-glycolic acid) (PLGA) gels with this pore size range proved to be suitable carriers for nucleus pulposus cells according to Kim and his coworkers [45]. It can be also observed (Fig 1c) that the pore size of the dried, solid gel matrices is almost independent from the chemical constitution of the hydrogels (Table 2). Regarding the two photon microscopy, PASP based polymer scaffolds have an auto-fluorescent activity [34] therefore there was no need to stain the gel samples before their investigation. The two photon images further emphasize that the physical appearances of the different hydrogel samples are similar (Fig 1c).

**Table 2:** Arithmetic mean ± standard deviation of pore size of dried hydrogels determined from SEM microscopic images.

	CYSE _(1/2)_	CYSE _(1/5)_	CYSE _(1/10)_	CYSE _(1/20)_	CYSE _(1/40)_	CYSE _(1/80)_
Poresize	66±9 μm	78±14 μm	78±18 μm	74±13 μm	73±15 μm	76±17 μm

The microscopic structure of the hydrogel undoubtedly affects cell proliferation. If the pore size of the hydrogels is higher, the cells are able to not only attach to the outer surface of the gel discs but also migrate inside the scaffold forming three dimensional structures. Thus, investigating the microscopic structure of the gels is essential. For characterization, two photon and SEM microscopies were implemented to assess and measure the size of the pores.

### Cell culturing on differently crosslinked poly(aspartamide) hydrogels

The mechanical and chemical properties (degradability, porosity, stiffness, binding points) of hydrogels have a significant impact on cell adhesion, viability and proliferation [27]. Among the aforementioned properties, stiffness is crucial. During adhesion, the cells receive mechanical feedbacks from their environment through mechanotransduction and respond by altering their cytoskeleton and morphology [46].

Mechanical properties of hydrogels can be easily tailored by the modification of the monomer unit/cross-linker ratio or by substituting the crosslinking agent. The exact parameters of these hydrogels were presented previously: the elastic moduli of the stiffer hydrogels (1/20) are between 55.3 and 66 kPa while the moduli of the softer gels (1/40) are between 7.2–10.5 kPa [34]. Our results are in accordance with the outcomes described by Engler and coworkers [46]. When the aim is neurogenic differentiation, the use of soft gels (0.1 to 1 kPa) is recommended, whereas in the case of myogenic direction, higher stiffness (8 to 17 kPa) is the optimal, finally for osteogenic differentiation, rigid matrices (25–40 kPa) are most suitable.

**Fig 2:**
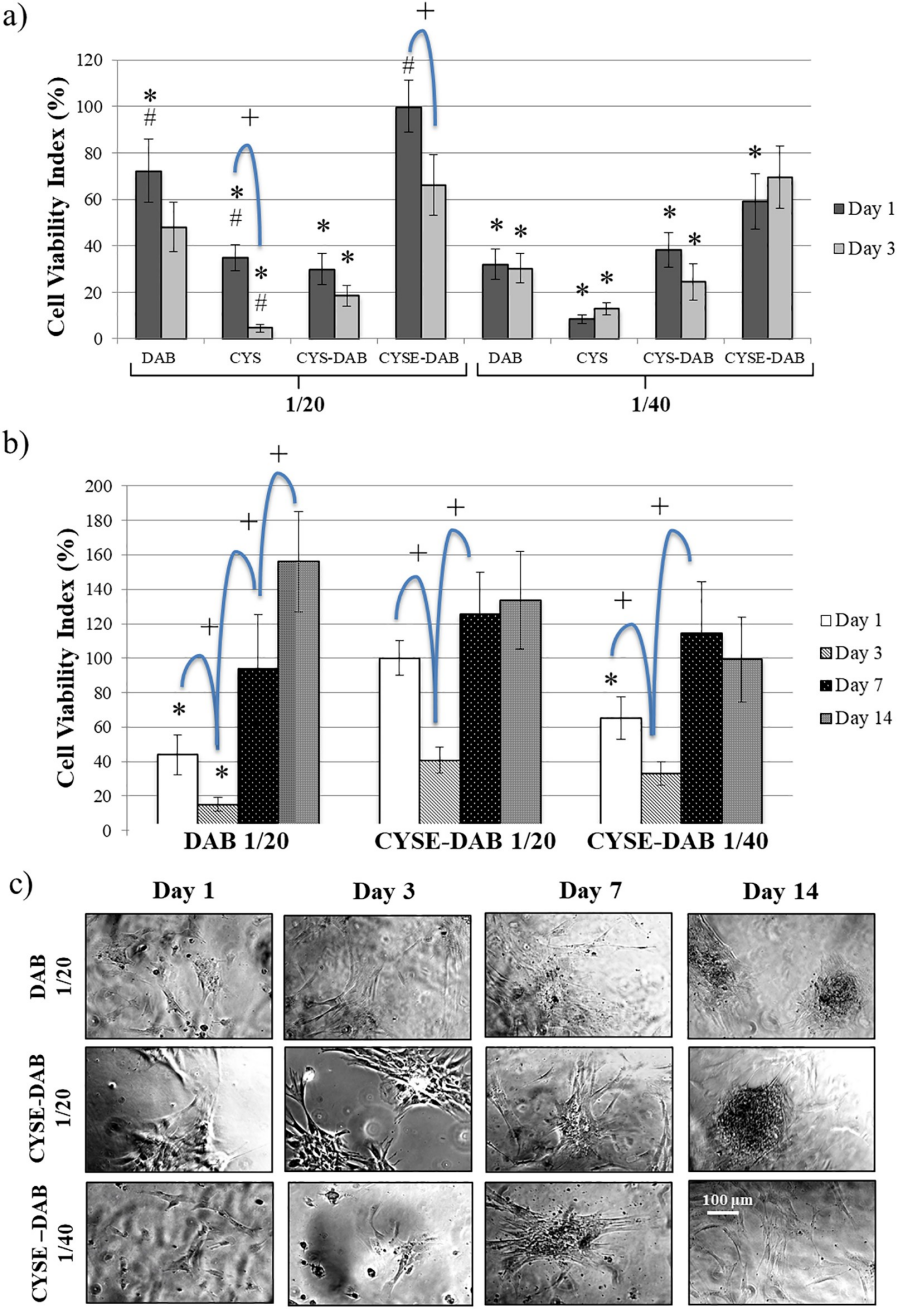
Cell viability on different PASP hydrogels on day 1 and 3 (a), long term viability on PASP-DAB_1/20_, CYSE-DAB_1/20_ and CYSE-DAB_1/40_ gels (b) and phase-contrast microscopic images of PDLCs in long term experiments at different time points (c). The viability value measured on CYSE-DAB_1/20_ gels on day 1 was considered 100%. Data are given as an arithmetic means ± SEM (standard error of the mean). *p < 0.05 compared to CYSE-DAB1/20 at the appropriate time point. +p < 0.05 compared to the next day. Each photomicrograph was taken at the same magnification.

At first we focused on the mechanical properties of the different hydrogels. In Fig 2a, cell viability results are presented after 1 and 3 days on 8 different PASP-based gel types. After 24 hours, the highest cell viability values can be observed on DAB_1/20_ and CYSE-DAB_1/20_ gel discs. The cell viability index of CYSE-DAB_1/20_ is significantly higher than that of the other gel types suggesting that a stiffer gel surface and the presence of free thiol groups can facilitate the adhesion of PDLCs. Stiffness of the microenvironment does not only affect cell adhesion, but migration and differentiation as well. Khatiwala and coworkers demonstrated similar results regarding stiffer surfaces, where MC3T3-E1 preosteoblasts exhibited increased proliferation, motility and mineral deposition compared to softer scaffolds [47]. It seems that scaffold stiffness strongly influences the cell lineage direction, although during the initial differentiation period alteration of the lineage with soluble induction factors is still possible. Our results regarding increased adhesion and proliferation of PDL cells on stiffer hydrogel surfaces correspond with the aforementioned studies on preosteoblast cells [47].

Similar results are found after 72 hours, when the stiffer DAB-crosslinked and two thiolated (CYSE-DAB_1/20_ and CYSE-DAB_1/40_) hydrogels evince the highest cell viability values. All other gel types show significantly lower viability values than the CYSE-DAB_1/20_. According to these findings, the second step was to focus on the effect of the thiol groups. The presence of free thiol groups has a positive influence not only on cell adhesion but on survival and proliferation of PDL-derived cells as well. In addition, these observations are confirmed by the results of the phase-contrast microscopic studies (S3 Fig). However, on certain gel types (CYS_1/20_ and CYSE-DAB^1/20^), significantly lower cell viability was found at day 3 compared to day 1. To clarify the reason behind this decrease and further analyze the long-term biocompatibility of the gels, 14 day-long experiments were also carried out.

The three hydrogel types that exhibited the highest cell viability during the first experimental series were chosen for a second, 14-day-long series of experiments. The results of this assay can be seen in Fig 2b. On day 1, the highest cell viability index was measured on the stiffer, thiol containing gel. Between days 1 and 3, a tendency of descending viability was observed on all 3 gel types. However, a significantly increased viability was observed by day 7 compared to day 3 on each gel type. This rising tendency continues until day 14 at the DAB_1/20_ gel but no further changes can be observed after day 7 in case of the thiol containing gels. By day 14, the viability of PDL cells reaches similar level on all of the 3 gel types. Therefore, the favorable effect of thiol content is more prominent in the phase of adhesion (day 1) and during the proliferation at lower cell density (day 3–7). The descending tendency between days 1 and 3 can be explained by the fact that the PDLC cultures represent a mixed population, and probably only the adhesion of a subpopulation is selectively promoted by the mechanical properties of the gels [47]. Subsequently, proliferation of this subpopulation enhanced but growth of the other cells is not supported by the mechanotransductional signals [48]. The aforementioned results indicate that these 3 types of PASP based gels remain stable and biocompatible in the long term as well.

The morphological analysis carried out under a phase contrast microscope (Fig 2c) shows a relatively small amount of fibroblast-like cells on the gel surfaces on days 1 and 3. After that, the cell number is increasing on the stiffer gels with time. By the 14^th^ day of culture, cell aggregations were also found on these gels, which suggests a spontaneous osteogenic activity. On the contrary, fewer cells can be observed on the softer, CYSE-DAB_1/40_ gel type where no proliferation took place after the 7^th^ day.

**Fig 3:**
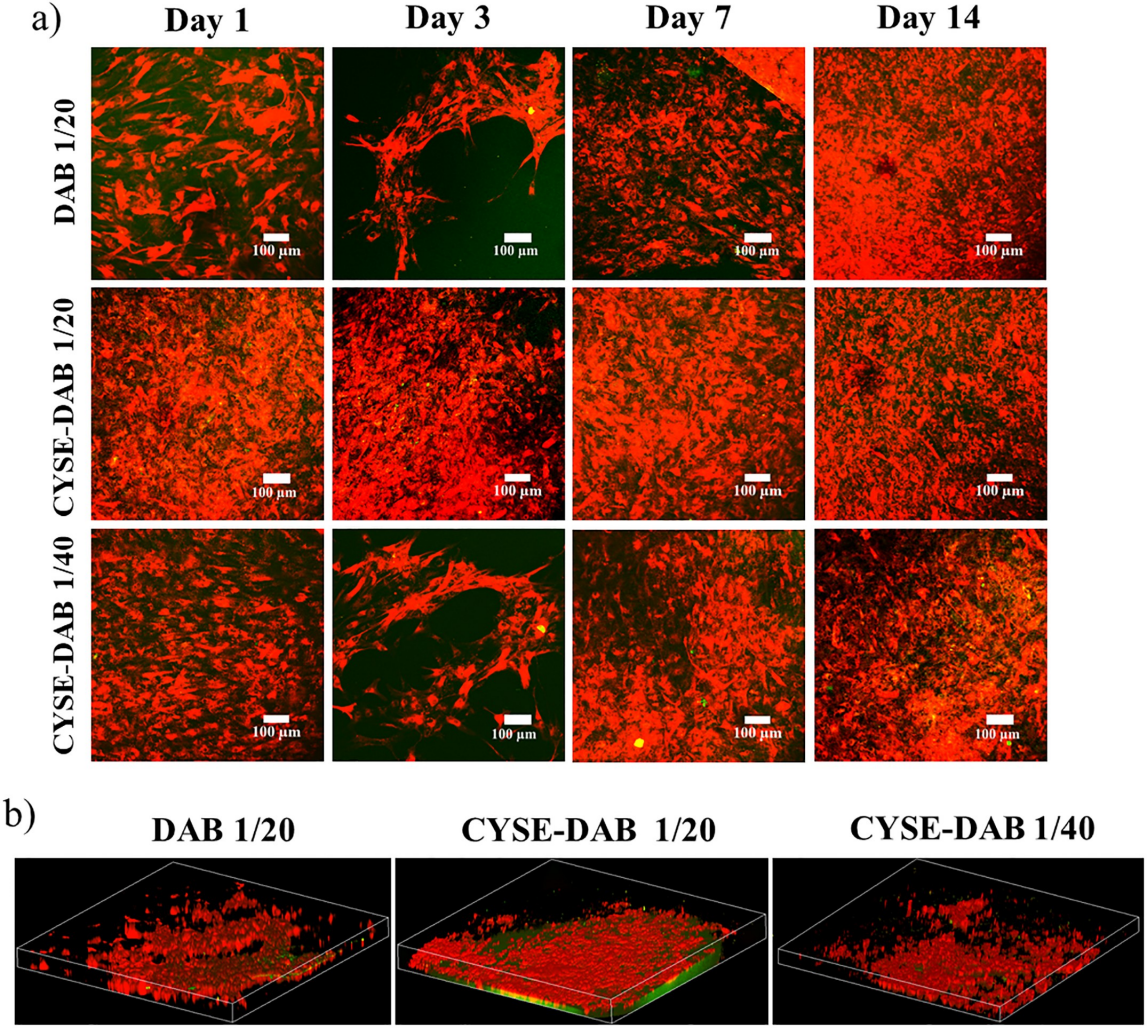
a) Two photon microscopic images of PDLC cultures in long term experiments at different time points and b) 3 dimensional structure of the cells in the hydrogels after 14 days. The Z depth of the 3D images indicates 120 μm, while the area is 1.7x1.7 mm.

To investigate the 3D morphology of the cells and matrix-cell relationship, a two-photon microscope was utilized. Since PASP-based hydrogels show a characteristic green autofluorescence and PDL cells reflect a red color due to the vital pre-staining, the cells can be easily distinguished from the gel matrix. On day 1, the highest amount of healthy cells was observed on the CYSE-DAB_1/20_ gel (Fig 3a). By day 3, a temporary decrease in cell number was found, whereas a remarkable cell proliferation was observed on all 3 gel types subsequently (Fig 3a).

In Fig 3b, the three-dimensional structure of the gels can be seen after 14 days. These images and the video supplement clearly demonstrate that the cells were able to penetrate into the gel matrix.

The microscopic analysis in our study verified that stiffer and thiolated PASP gels are indeed more suitable for cell culturing, as PDLCs on these gels exhibited healthy, fibroblast-like morphology and intensive proliferation. The two-photon microscopic pictures demonstrated that PDLCs are able to grow into the gel matrix. The penetration of the cells into the gel matrix may provide indirect evidence of the in vitro biodegradability of the PASP hydrogels. According to our previous article [34] and Fig 1, there are two possible ways for cells to migrate in the gel matrix: either to liberate space by degrading the gel structure and/or penetrate by crawling over the walls of the pores. Presumably, both processes are going parallel and take part in vertical cell migration to a similar extent. Our results are in line with the work published by Matsusaki and coworkers who succeeded in constructing dense 3D tissues from mouse fibroblast cells cultured on disulfide-crosslinked polyglutamic acid hydrogels. Furthermore, positive effect of thiol groups on cell adhesion and proliferation was also confirmed in that study [49].

### Effects of thiol content on cell behaviour

#### Morphological analysis and long-term cell viability

The first two experimental series revealed that the presence of thiol groups in the gel supports adhesion of PDLCs and their proliferation especially in long terms (7–14 days). These results are consistent with a recent publication by Galli and coworkers demonstrating that enrichment of thiol content in chitosan scaffolds enhanced proliferation and migration of osteoblast cells [18]. These observations could be explained by the hypothesis of several studies conjecturing that charged molecules, such as thiol groups enhance the surface potential of scaffolds therefore have a positive impact on cell adhesion and proliferation [50, 51].

The aim of our third series of experiments was to address the question if the density of thiol groups in the gel matrix has any effect on the adhesion and proliferation of PDLCs. In these experiments, 6 hydrogel types having the same degree of crosslinks yet with increasing thiol group density (from (1/80) to (1/2)) were examined. The CYSE_(1/20)_ gel was used as a reference.

**Fig 4:**
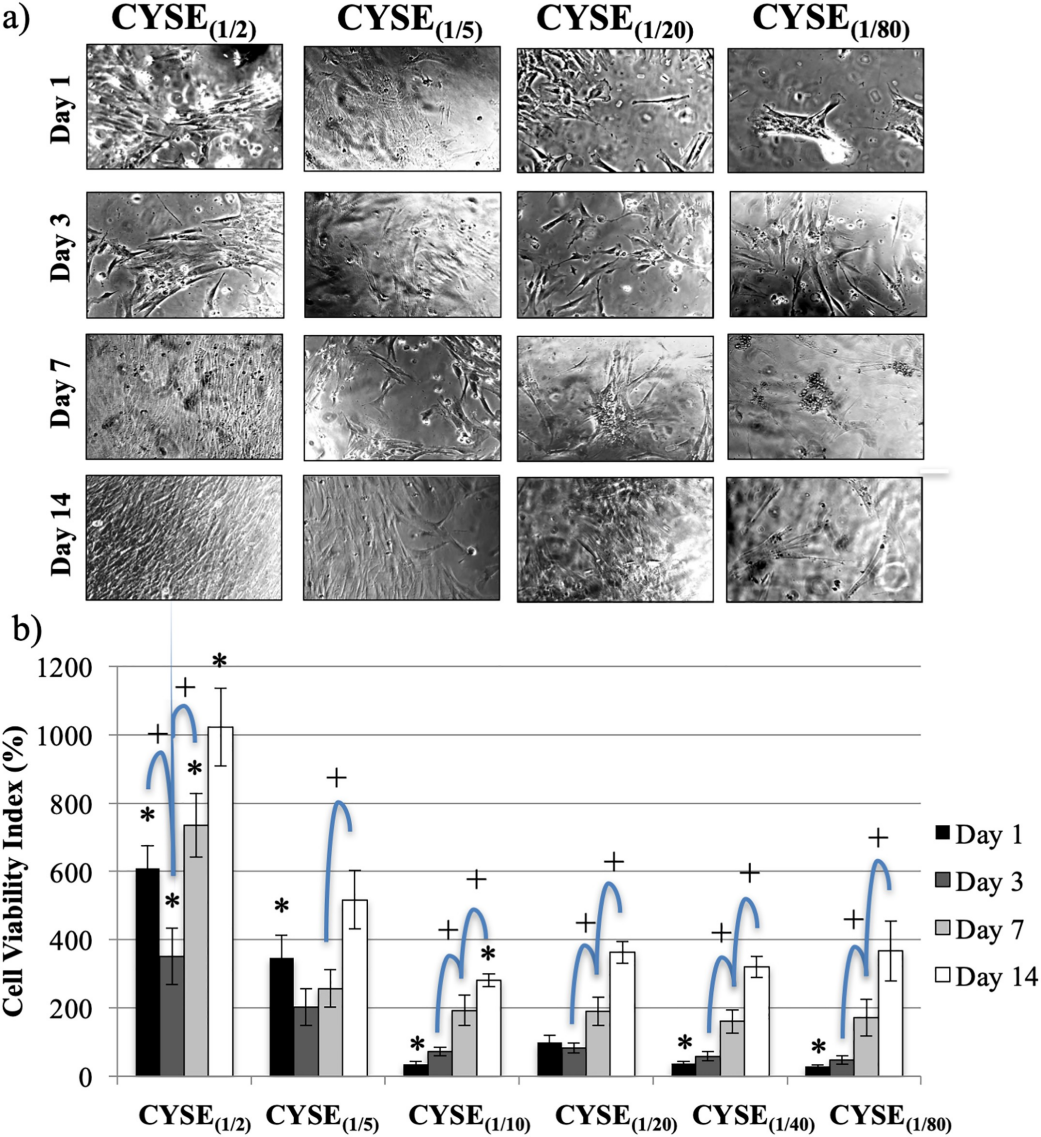
a) Phase-contrast microscopic pictures of PDL cells on PASP hydrogel discs containing different quantity of thiol groups. b) PDLC viability measured 1, 3, 7 and 14 days after seeding. The average viability value measured on the CYSE_(1/20)_ gels on day 1 was considered as 100%. Data are given as an arithmetic mean ± SEM (standard error of the mean). *p < 0.05 compared to CYSE(1/20) at the appropriate time point. +p < 0.05 compared to the next day values. Each photomicrograph was taken at the same magnification. The scale bar indicates 100 μm.

Morphological analysis with phase-contrast microscope showed a tendency of healthy, proliferating, fibroblast-like cells in increasing amount with the rising density of thiol-groups in the gels (Fig 4a). The number of healthy cells remarkably increased over time then it reached a confluent state by day 14 on both CYSE_(1/2)_-DAB and CYSE_(1/5)_-DAB samples. However, cell proliferation to a smaller extent was observed on the other gel types with lower thiol group densities. These results indicate that the increased density of thiol groups to more than 1/10 has not only a transient but rather a prolonged positive influence on cell growth.

Cell viability results at time points of morphological evaluation on six different gel types are presented in Fig 4b. After 24 hours, the two gels with the highest thiol group densities showed the highest cell viability values. On these two gel types, a temporary decrease in viability can be observed between day 1 and 3 followed by a significant increase until the end of the experiment. Although the initial cell viability is somewhat lower on the four other gel types, continuously ascending viability values are observed throughout the whole experiment. These measurements confirm that the high density of thiol groups in these gels provides favorable conditions for the adhesion and proliferation of PDL cells.

#### Investigation of spontaneous and induced osteogenic differentiation

We demonstrated that the presence of thiol groups undoubtedly had a positive effect on the adhesion and proliferation of the PDLCs.

Bae and coworkers determined through in vivo experiments that BMP-2 containing thiolated chitosan scaffolds induced more ectopic bone formation in mouse osteoblast cells than the BMP-2 containing collagen gels [52]. We presume that the influence of these thiol groups is related to the release of hydrogen sulfide, an intracellular gaseous signaling molecule involved in osteogenic differentiation [53, 54]. To clarify this hypothesis, we performed a parallel investigation of spontaneous and induced osteogenic differentiation of the PDLCs on thiolated PASP based hydrogels.

PDLCs were maintained in either a control or an osteogenic medium for 14 days. Osteogenic activity was followed by the measurement of alkaline-phosphatase activity. These experiments were carried out with the three gels showing the best results in the previous experiments: CYSE_(1/2)_, CYSE_(1/5)_ and CYSE_(1/20)_ while the DAB_1/20_ gel was applied as a non-thiolated control gel.

**Fig 5:**
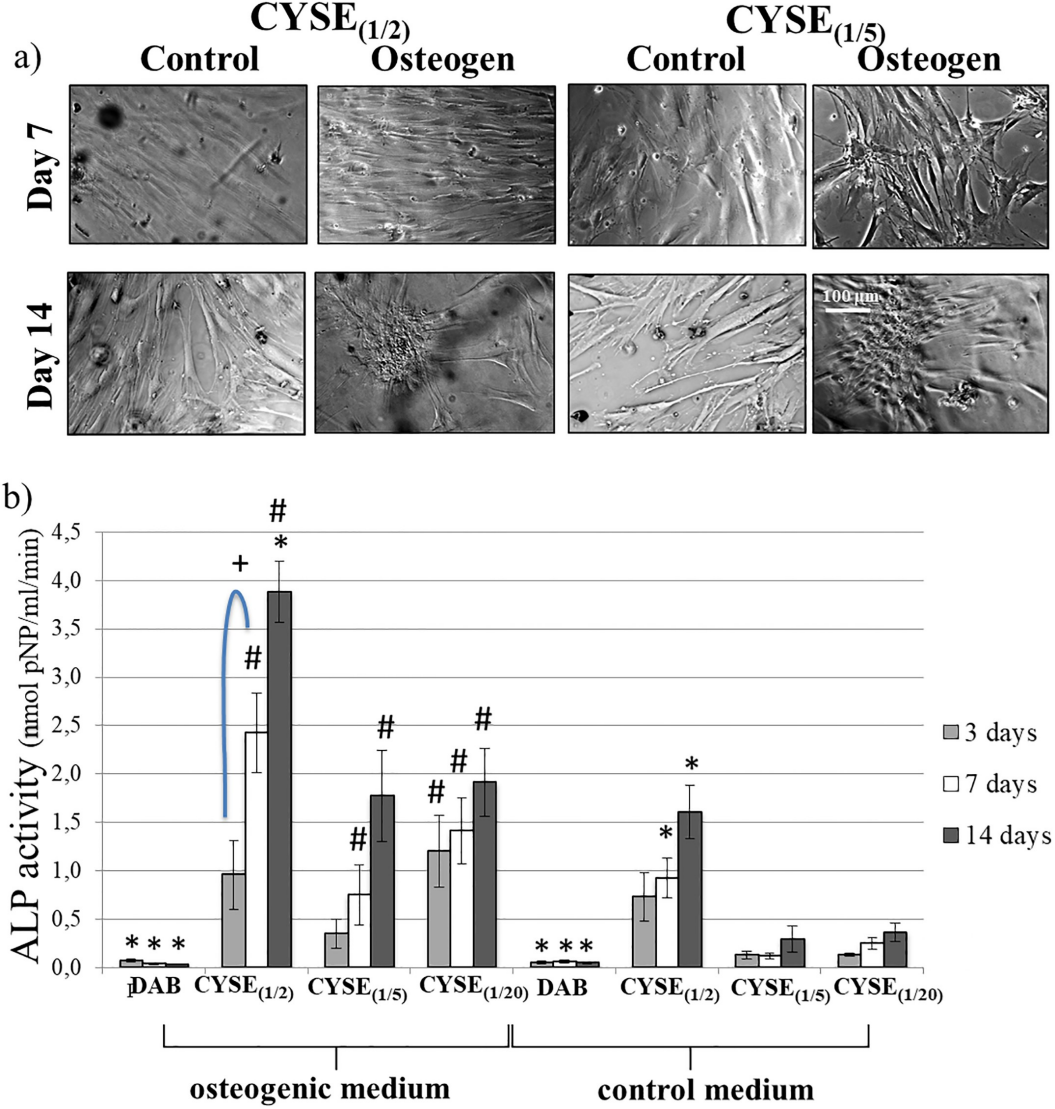
a) Phase-contrast microscopic pictures of osteogenic inducted and control PDL cells on PASP hydrogel disks containing different quantity of thiol groups. b) ALP activity of PDL cells cultured in osteogenic or control medium on PASP hydrogel disks after 3, 7 and 14 days. Each photomicrograph was taken at the same magnification. The scale bar indicates 100 μmData are given as an arithmetic mean ± SEM (standard error of the mean). *p < 0.05 compared to CYSE(1/20) at the appropriate time point. +p < 0.05 compared to next day values. #p < 0.05 compared to the appropriate control gel values.

PDLCs exhibited a healthy, fibroblast-like morphology on every gel type throughout the observation period. In the osteogenic medium, cell cluster formation could be detected after 14 days (Fig 5a). Fig 5b shows that the osteogenic treatment induced a measurable ALP activity in the thiol-containing gels with an increasing tendency in values over time. Although ALP activity at the 3 types of thiol-containing gels was similar on day 3, cell cultures on CYSE_(1/2)_ gels reached significantly higher ALP activity levels compared to the others by day 14. However, the cells on the DAB-crosslinked gel exhibited no osteogenic activity throughout the whole experimental period. The presumable explanation of this observation is that the PDL cells could not reach high cell density on these gels which is a prerequisite for starting the osteogenic differentiation process [55]. In the control medium, spontaneous osteogenic activity was detected at all thiol-containing gel types as early as on day 3 which further increased later on until day 14. Nevertheless, no sign of such activity could be observed in the case of the DAB-crosslinked gels throughout the whole experiment. The highest spontaneous osteogenic activity was measured in the case of the highest thiol density (CYSE_(1/2)_) at each time point. The range of the ALP activity values measured in case of CYSE_(1/2)_ gels were found to be similar to the ALP activity of the same cell type cultured on a commercially available peptide hydrogel called HydroMatrix [56].

**Fig 6:**
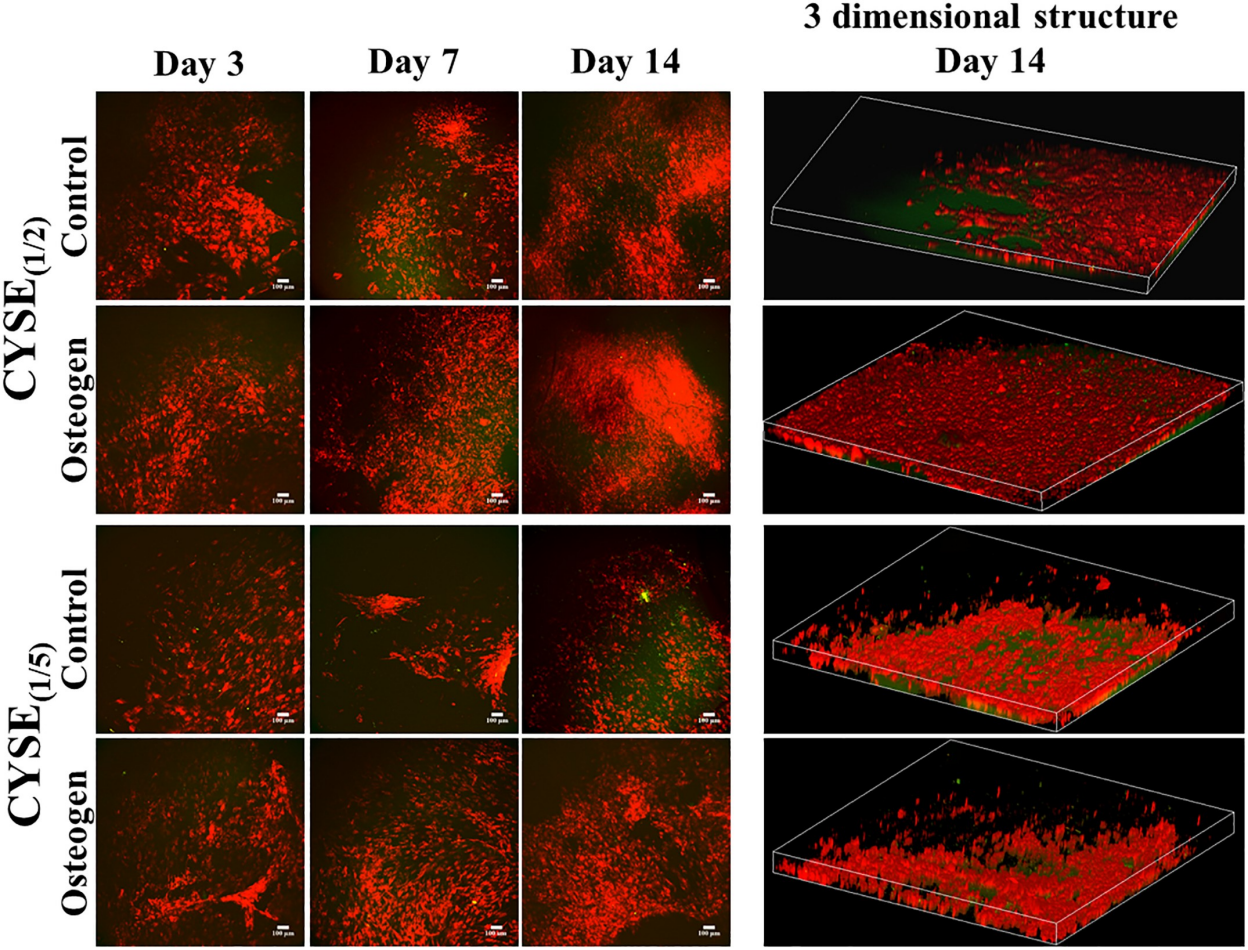
a) Two photon microscopic images of control and osteogenic induced PDLC cultures in long-term experiments at different time points and b) 3 dimensional structure of the cells in the hydrogels after 14 days. The Z depth of the 3D images indicates 120μm while the area is 1.7x1.7 mm.

The two-photon microscopic analysis (Fig 6a) underlined the above described results regarding phase contrast microscopic morphology (Fig 4a) and cell viability tests (Fig 4b). The highest cell density could be observed on the hydrogels with the highest amount of thiol-groups. By stacking the photos of different heights, a 3D structure of the samples was reconstructed. This stacked 3D image provides evidence that PDLCs are steadily growing and migrating and spreading not only along the gel surface but inside the gel matrix (Fig 6b).

Therefore, the high amount of thiol groups in the gels supports the survival, adhesion, proliferation, migration and 3D growth of PDLCs.

## Conclusion

To the best of our knowledge, we are the first to investigate the behavior of tooth-derived cells on different thiolated PASP based hydrogels. To briefly summarize our results, poly(aspartic acid) based hydrogels proved to be considerably biocompatible and biodegradable. Furthermore, these hydrogels did not only support survival but adhesion, proliferation, and migration of human PDLCs as well, especially when free accessible thiol groups were present. The increased amount of free thiol groups in the gel matrix resulted in significantly higher cell viability and facilitated spontaneous osteogenic differentiation of PDLCs. Therefore, these thiolated PASP-based hydrogels seem to be ideal scaffolds for culturing and differentiating human tooth-derived cells. However, further preclinical animal experiments are needed before these hydrogels could be potentially applied in different areas of regenerative therapy.

## Supporting information

S1 Table Constitution of the reaction mixtures. Preparation of poly(succinimide) gels with different cross-linkers and cross-linking ratio.
(PDF)

S2 Table Constitution of the reaction mixtures. Preparation of poly(succinimide) gels with different thiol contents.
(PDF)

S1 Fig Schematic figure of the hydrogel synthesis with different thiol content. (TIF)

S2 Fig Calibration for thiol groups measurements. (TIF)

S3 Fig Phase-contrast microscopic pictures of PDL stem cells on PASP hydrogel disks with different cross-linking agents (DAB or CYS) and crosslinking degree 20 or 40. Each photomicrograph was taken at the same magnification. The scale bar indicates 100 μm.
(TIF)
