# Supplementary material for: Free thiol groups on poly(aspartamide) based hydrogels facilitate tooth-derived progenitor cell proliferation and differentiation
Source: PLoS One. 2019 Dec 19;14(12):e0226363. doi: 10.1371/journal.pone.0226363 (PMC6922333; doi:10.1371/journal.pone.0226363)
Supplement: S1 Table — Preparation of poly(succinimide) gels with different cross-linkers and cross-linking ratio. (PDF) [file pone.0226363.s001.pdf]

Table S1: Constitution of the reaction mixtures

Sample name	25 w% PSI solution (mg)	DAB (mg)	CYS*2HCl (mg)	DBA (mg)	DMSO (mg)
DAB 1/20	600.0	6.8	-	-	393.2
CYS 1/20	600.0	-	17.4	20.0	362.6
CYS-DAB 1/20	600.0	3.4	8.7	10.0	377.9
CYSE-DAB 1/20	600.0	6.8	17.4	20.0	355.8
DAB 1/40	600.0	3.4	-	-	396.6
CYS 1/40	600.0	-	8.7	10.0	381.2
CYS-DAB 1/40	600.0	1.7	4.4	5.0	388.9
CYSE-DAB 1/40	600.0	3.4	8.7	10.0	377.9

DBA: Dibutylamine

DMSO: Dimethyl sulfoxide
