# Supplementary material for: Free thiol groups on poly(aspartamide) based hydrogels facilitate tooth-derived progenitor cell proliferation and differentiation
Source: PLoS One. 2019 Dec 19;14(12):e0226363. doi: 10.1371/journal.pone.0226363 (PMC6922333; doi:10.1371/journal.pone.0226363)
Supplement: S2 Table — Preparation of poly(succinimide) gels with different thiol contents. (PDF) [file pone.0226363.s002.pdf]

Table S2: Constitution of the reaction mixtures

Sample name	25 w% PSI solution (mg)	DAB (mg)	CYSE (mg)	DMSO (mg)
$CYSE_{(1/2)}$	600.0	6.8	59.5	333.7
$CYSE_{(1/5)}$	600.0	6.8	23.8	369.8
$CYSE_{(1/10)}$	600.0	6.8	11.9	381.2
$CYSE_{(1/20)}$	600.0	6.8	5.9	387.2
$CYSE_{(1/40)}$	600.0	6.8	2.9	390.2
$CYSE_{(1/80)}$	600.0	6.8	1.5	391.7
