# Supplementary figures and images for: Free thiol groups on poly(aspartamide) based hydrogels facilitate tooth-derived progenitor cell proliferation and differentiation
Source: PLoS One. 2019 Dec 19;14(12):e0226363. doi: 10.1371/journal.pone.0226363 (PMC6922333; doi:10.1371/journal.pone.0226363)

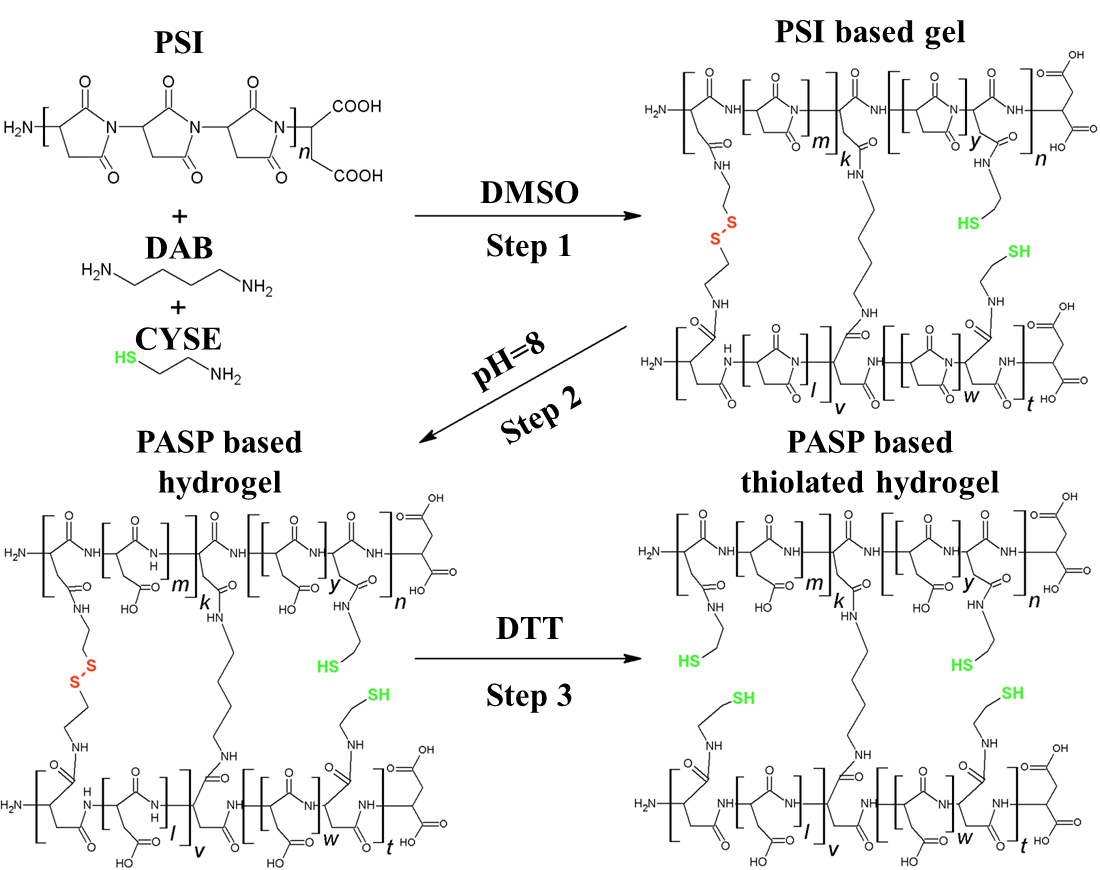

Supplement: S1 Fig — (TIF) [file pone.0226363.s003.tif]

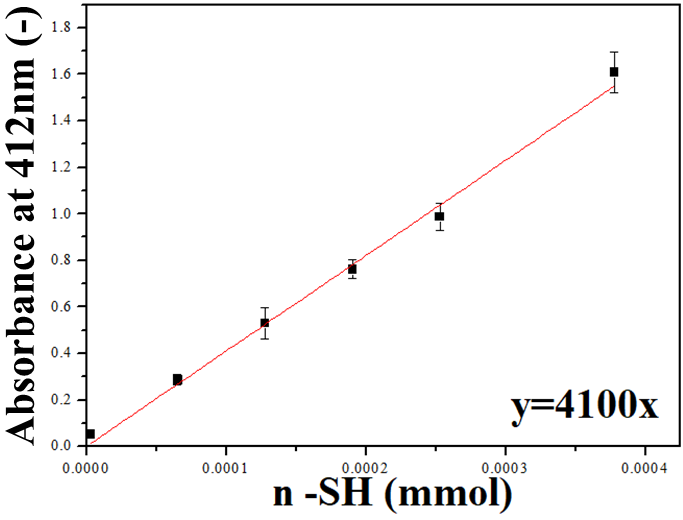

Supplement: S2 Fig — (TIF) [file pone.0226363.s004.tif]

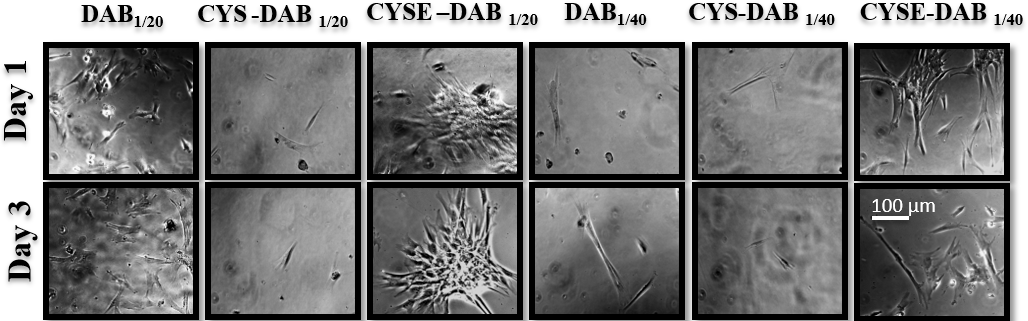

Supplement: S3 Fig — Each photomicrograph was taken at the same magnification. The scale bar indicates 100 μm. (TIF) [file pone.0226363.s005.tif]
